# Antibiotic prescriptions and cycles of *Mycoplasma pneumoniae* infections in Norway: can a nationwide prescription register be used for surveillance?

**DOI:** 10.1017/S0950268814002908

**Published:** 2014-11-12

**Authors:** H. S. BLIX, D. F. VESTRHEIM, V. HJELLVIK, D. SKAARE, A. CHRISTENSEN, M. STEINBAKK

**Affiliations:** 1 Department of Pharmacoepidemiology, Norwegian Institute of Public Health, Oslo, Norway; 2 School of Pharmacy, University of Oslo, Norway; 3 Department of Bacteriology and Immunology, Norwegian Institute of Public Health, Oslo, Norway; 4 Department of Microbiology, Vestfold Hospital Trust, Tønsberg, Norway; 5 Department of Medical Microbiology, St. Olavs Hospital, Trondheim University Hospital, Trondheim, Norway

**Keywords:** Antibiotics, community epidemics, epidemiology, public health, surveillance system

## Abstract

*Mycoplasma pneumoniae* outbreaks cause increased use of macrolides and tetracyclines. We aimed to investigate whether drug use data, in addition to laboratory data, could improve understanding of the spread of *M. pneumoniae* epidemics. Number of users of *Mycoplasma* antibiotics (erythromycin, doxycycline, clarithromycin) per week and county of residence in an indicator age group (6–12 years) was retrieved from the Norwegian prescription database for the epidemic season 2011–2012 and compared to non-epidemic seasons. In 2011, increased use of *Mycoplasma* antibiotics was first observed in September on the west coast of Norway. The Norwegian laboratory-based surveillance system showed the first increase in positive tests in August 2011 and an epidemic was announced on 25 October 2011. At that time the use of *Mycoplasma* antibiotics had already exceeded three times the use in non-epidemic periods. Data for three counties from the regional microbiological laboratories showed that the increase in number of positive samples coincided in time with the increase in prescription data. Laboratory data cannot accurately determine the extent of an epidemic, and drug use data cannot identify the cause. Establishing a systematic interaction between the two monitoring systems will enhance surveillance and probably contribute to improved infection control and prudent antibiotic prescribing.

## INTRODUCTION

*Mycoplasma pneumoniae* is a common cause of respiratory tract infections. Clinical symptoms are mostly mild and may vary from asymptomatic to severe disease. Attachment of *M. pneumoniae* to host cells in the respiratory tract is a prerequisite for colonization and infection. Cytoadherence, mediated by the P1 adhesin and other accessory proteins is followed by a chronic inflammation with symptoms of prolonged cough [1, 2]. Antibiotics have limited effect on the duration of symptoms, and should be reserved for severe disease [3]. Epidemics of *M. pneumoniae* infections occur in 5- to 7-year cycles. In years with epidemics of *M. pneumoniae*, waves of increased use of macrolides and tetracyclines are observed [4]. The time window for these waves is often broader than the time window for the epidemic, as reported by the microbiological laboratories, indicating a possible temporary change in physicians' prescribing behaviour.

Overuse of antibiotics causes antibiotic resistance and many countries implement antibiotic stewardship programmes to try to achieve appropriate prescribing [5, 6]. In ambulatory care, prescribing is often empirical, based on the clinical picture and not on the aetiological agent. Awareness of an ongoing epidemic will influence the empirical choice of drug treatment. It is therefore important that the health authorities provide prescribers with up-to-date information about ongoing epidemics; at the beginning of the epidemic, and preferably also when the epidemics are fading out. The latter is important to reduce unnecessary prescribing of antibiotics that do not comply with prescribing guidelines. *M. pneumoniae* epidemics are not generally a threat to public health, but antibiotic resistance is, and because the antibiotics prescribed for *M. pneumoniae* are broad-spectrum in the sense of having increased potential for antimicrobial resistance, it is a task for the public health authorities to give advice for rational prescribing [7].

*M. pneumoniae* infections are not notified to the Norwegian Surveillance System for Communicable Diseases, but epidemics can be observed through a voluntary laboratory-based surveillance system, relying on positive laboratory results. However, this voluntary surveillance cannot capture the full picture of the epidemic, and is prone to bias caused by diagnostic testing activity. The microbiological laboratories are not equipped to analyse large numbers of samples, so when an epidemic is identified, physicians are often urged not to send more samples, since additional analyses will not add new information about the epidemic. So although the voluntary laboratory-based surveillance system is good for identifying an upcoming epidemic, it is not equally well suited to follow the course of the epidemic. Drug use data could possibly be a data source giving a more detailed picture of the epidemic.

In autumn 2011, an epidemic of *M. pneumoniae* was observed in Norway, as well as in other Nordic and Northern European countries [8–10]. We aimed to investigate if data from a drug prescription database could be used to help in describing the epidemic and whether the drug use data could add more information to the voluntary laboratory-based surveillance system of epidemics.

## METHODS

### Data on antibiotic use

Data on antibiotic use for all individuals living in Norway were retrieved from the Norwegian prescription database (NorPD). In the NorPD, data on prescribed medicines from all persons living in Norway have been collected since 2004. The identity of patients is encrypted, and through a unique personal identification number it is possible to identify prescriptions dispensed at Norwegian pharmacies to individuals living outside health institutions. The NorPD contains demographical information, such as age, gender, place of residence and information on all prescribed drugs, reimbursed or not. In Norway, all antibiotics are prescription-only drugs which implies that all sales of antibiotics are captured by the NorPD. The drugs are classified according to the Anatomical Therapeutic Chemical (ATC) classification system [11].

According to Norwegian guidelines, erythromycin (ATC code J01FA01) or doxycycline (ATC code J01AA02) are recommended as drugs of choice if treatment of respiratory tract infections caused by *M. pneumoniae* is needed [12]. Further, clarithromycin (ATC code J01FA09) is recommended as second-line treatment. Azithromycin and spiramycin, the only other macrolides available in Norway, are not recommended for *M. pneumoniae* in Norway. These three antibiotics (erythromycin, clarithromycin, doxycycline) are hereafter referred to as *Mycoplasma* antibiotics.

Data were drawn from the NorPD for all individuals who had received *Mycoplasma* antibiotics in the period 1 January 2004 to 31 December 2012. Parameters included in this study were: each patient's unique identification number (encrypted), gender, age, county of residence, date of dispensing, drugs with ATC code (J01FA01, J01FA09, J01AA02) and, for patients who had died, the month of death.

We counted for each week the number of persons – in 1-year age groups – who were dispensed at least one *Mycoplasma* antibiotic during the week. Week 1 was defined as 1–7 January each year. Weeks 52 and 53 were excluded due to many public holidays with closed pharmacies (week 52 started 24 December in non-leap years). To calculate the weekly number of users per 1000 inhabitants, we interpolated the weekly number of inhabitants from the population per county and age by 1 January each year.

### Data from laboratories

In Norway, a total of 21 medical microbiological laboratories analyse data on human samples. National data on results of microbiological respiratory tract samples testing positive for *M. pneumoniae* are reported through the voluntary laboratory-based surveillance system. Since 2012, 16 laboratories in the country have participated in this reporting system, handled by the Norwegian Institute of Public Health. This system only collects positive test results and no data on total number of samples tested nor any demographic data on patients. We therefore invited the laboratories to supply us with more specific data. Two regional medical microbiological laboratories were able to deliver demographical data for all positive samples in three counties; two counties with extensive spread and one with little spread of the epidemic, for the period May 2011–March 2012. The information obtained was: age, gender, county of residence and date for receiving the sample. In January 2012, the two counties with extensive spread in southeast of Norway, Vestfold and Telemark, had 236 424 and 170 023 inhabitants, respectively, while the county with a less pronounced epidemic, Sør-Trøndelag, is situated in mid-Norway and had 297950 inhabitants.

Polymerase chain reaction (PCR) tests have been recommended as the most specific method of choice for laboratory diagnosis of suspected *M. pneumoniae* infections from 2003, since then the proportion of reported cases identified by PCR has increased, while the proportion reported by serology has decreased. In Vestfold and Telemark only PCR is used for the report whereas around 13% of reported cases from Sør-Trøndelag were identified by serology. To be consistent we have included only positive results from PCR in the analysis.

*Bordetella pertussis* infections are treated with the same antibiotics as *M. pneumoniae*. Since 1993, all cases of *B. pertussis* have been mandatory notifiable to the Norwegian Surveillance System for Communicable Diseases (MSIS) and to control for possible bias we also collected data on reported positive samples of *B. pertussis* [13].

Monthly prescription data were compared with monthly national data from the voluntary laboratory-based surveillance system. Furthermore, the specific data obtained from the three counties were compared with prescription data in the same counties.

### Identification of an indicator group

Antibiotic use in different age groups varies by amounts and types of antibiotics. There are seasonal fluctuations, with higher use in winter and lower in summer. Within the same age group the pattern and level of use is reasonable stable from one year to another. We aimed to identify a patient group that could possibly act as an indicator through *M. pneumoniae* epidemics, when using prescription data. The relative change in antibiotic use (i.e. deviation from baseline) during years with epidemics compared to years without epidemics is expected to be largest in age groups with a low use of antibiotics. In the 6–15 years age group, i.e. children in primary and secondary school, we found the lowest annual prevalence of antibacterial use [14]. A higher antibiotic use than normal in this indicator age group can be thought to indicate an epidemic.

To identify the optimal indicator group, we compared the use of *Mycoplasma* antibiotics during the epidemic in 2011–2012 with the three non-epidemic seasons 2008–2009, 2009–2010 and 2010–2011 in 1-year age groups, to determine which age groups had the highest amplitude. Thereafter, in order to simplify and to have a reasonable signal-to-noise ratio in less densely populated areas and for shorter periods of time, we grouped the adjacent ages with the highest amplitudes and used this as an indicator group.

### Geographical spread of epidemic and standardization/correction factors

Data in the NorPD are collected according to the place of residence at the community level. Data from the voluntary laboratory-based surveillance system is based on the patient's place of residence and reported at the county level. The population of the Norwegian communities ranges from very small (200 individuals) to large (600 000 individuals), and measurement of antibiotic use in the smallest communities is strongly influenced by the drug use of just a few individuals. To avoid such sensitivity and to be able compare prescription data with the voluntary laboratory-based surveillance data, patterns of use were described at the county level.

Prescription practice differs between counties, and to make the counties more comparable, a correction factor per county was computed as follows: The number of users per 1000 inhabitants by week and county was calculated for the non-epidemic seasons 2008–2009, 2009–2010 and 2010–2011. The average of the 153 weeks for each county was calculated, and divided by the corresponding average for the whole country. Prevalence estimates by week and county were then divided by this correction factor.

### Ethical approval

The study was based on anonymous patient data from laboratories and the patients' data in NorPD has an encrypted unique individual identification number (i.e. anonymous to the researcher). Therefore, there was no need of approval by an ethics committee.

## RESULTS

**Fig. 1.**
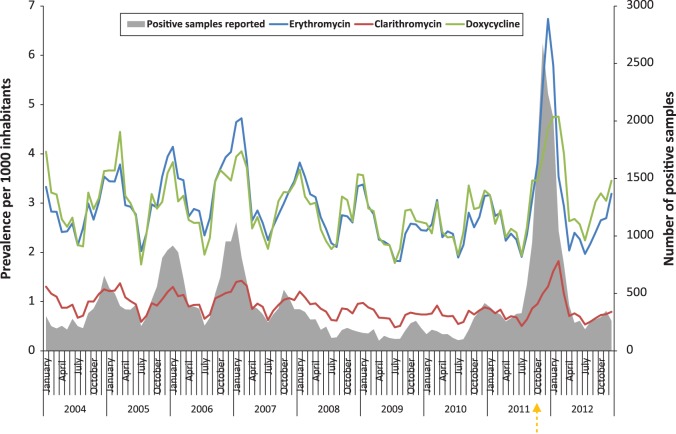
Prevalence of the total population in Norway (per 1000) using erythromycin, clarithromycin and doxycycline and number of positive tests for *M. pneumoniae* reported through the Norwegian voluntary laboratory-based surveillance system, 2004–2012. The orange arrow indicates the date the *M. pneumoniae* epidemic was announced from the Institute of Public Health (25 October).

In the population, the trend for use of chosen antibiotics is higher in winter and lower in summer. Clear peaks in prevalence are seen for the winter seasons of 2004–2005, 2005–2006, 2006–2007 and 2011–2012, coinciding with reports of *M. pneumoniae* epidemics from the voluntary laboratory-based surveillance system (Fig. 1). The use of *Mycoplasma* antibiotics increased particularly strongly during the winter season 2011–2012, erythromycin being the antibiotic showing the greatest fluctuation in use. The peaks were delayed for doxycycline (1 month) and clarithromycin (2 months) compared to erythromycin.

**Fig. 2.**
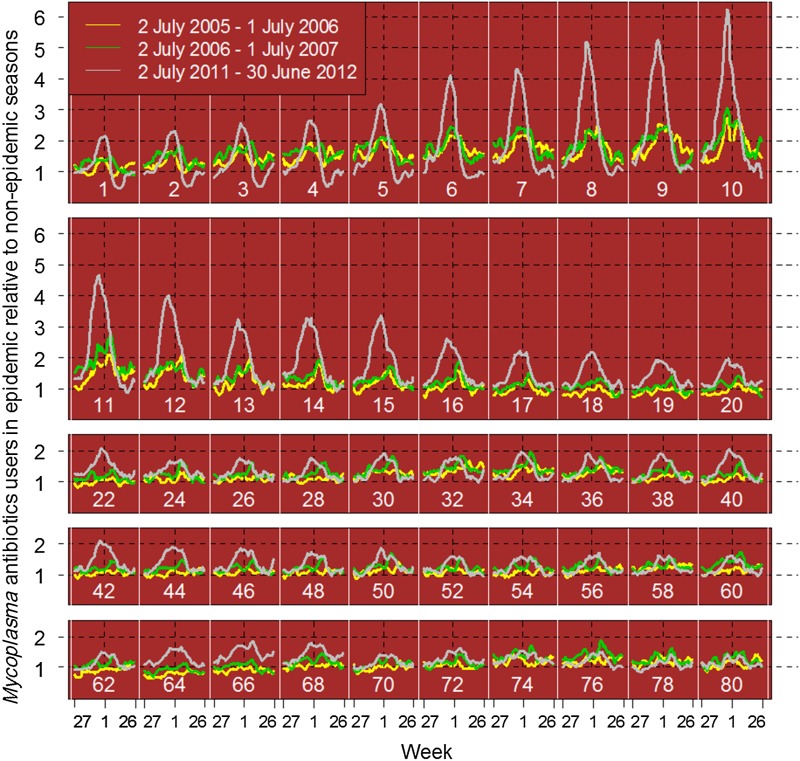
Deviations in use of *Mycoplasma* antibiotics (erythromycin, clarithromycin, doxycycline) per 1-year age group calculated as number of users of *Mycoplasma* antibiotics per week in the seasons 2005–2006 (yellow), 2007–2008 (green) and 2011–2012 (grey) divided by number of users per week averaged over seasons without *M. pneumoniae* epidemics, i.e. 2008–2009, 2009–2010, 2010–2011. White vertical lines and white numbers indicate age groups (age = *i*: was between *i* and *i* + 1 year when the antibiotic was dispensed). Each curve starts at week 27 and ends at week 26 the following year. Black vertical dashed lines indicate 1 January. Each point represents a moving average over 5 weeks (from 2 weeks before, to 2 weeks after the actual week).

The data, analysed in 1-year age groups, showed that the highest prevalence in 2011–2012 was seen in the youngest children. However, the largest deviation from baseline, i.e. relative to the use in the non-epidemic years, was found for the 6–12 years age group (Fig. 2). The same age pattern was seen in 2005–2006 and 2006–2007, but with smaller amplitudes. These ages were therefore merged and used further as an indicator age group.

**Fig. 3.**
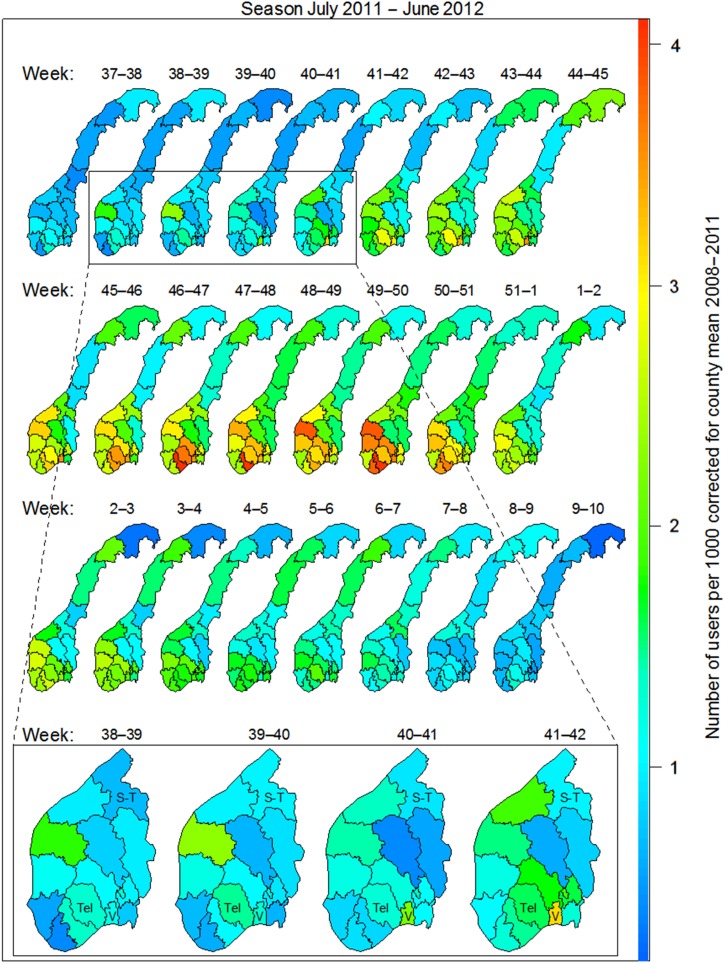
Users of *Mycoplasma* antibiotics (erythromycin, clarithromycin, doxycycline) per 1000 inhabitants in the 6–12 years age group per week and county in the 2011–2012 season. Corrected for county means in the non-epidemic seasons 2008–2011. Moving average over 2 weeks. Bottom panel: S-T, Sør-Trøndelag; Tel, Telemark; V, Vestfold.

**Fig. 4.**
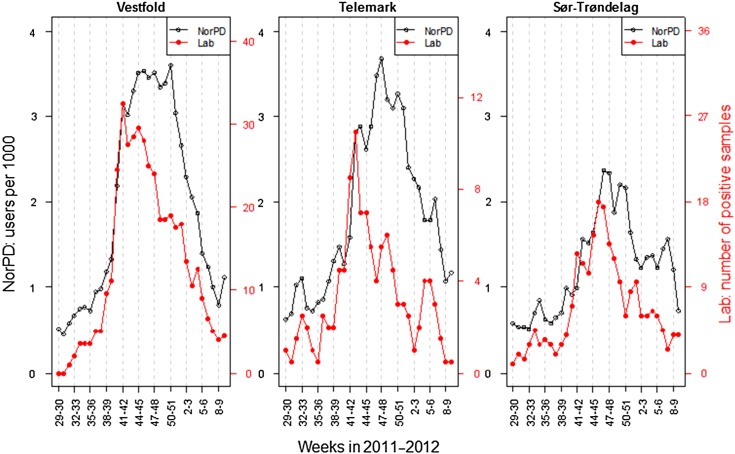
Number of positive samples of *M. pneumoniae* and users of *Mycoplasma* antibiotics (erythromycin, clarithromycin, doxycycline) per 1000 persons in children aged 6–12 years in three counties in Norway in 2011–2012. Moving average over 2 weeks.

The use of *Mycoplasma* antibiotics in the chosen indicator age group (6–12 years) was analysed per week according to county of residence (see Fig. 3). The figure shows the spread of the epidemic as measured by number of individuals per 1000 inhabitants that had *Mycoplasma* antibiotics prescribed, by time and county, corrected for county-level in non-epidemic seasons. Increased use was first observed on the west coast in weeks 38–39 (late September), in the county of Sogn og Fjordane. Here the use levelled off, but increased again in weeks 42–43. The most affected area is seen in the southeastern region of Norway. Increased use (above noise ratio) was observed in weeks 39–40 in Vestfold county and weeks 41–42 in the neighbouring county of Telemark (Fig. 3). Use of *Mycoplasma* antibiotics for the chosen indicator age group reached its highest level in Vestfold county in week 41 (mid-October 2011), week 46 in Sør-Trøndelag county and week 47 in Telemark county (Fig. 4). The eastern and northern parts of Norway appeared to be much less affected than the west and south. By the end of February 2012, the epidemic seemed to have faded out throughout the country.

The national data from the voluntary laboratory-based surveillance system showed the first increase in positive tests results in August 2011. The epidemic was announced on 25 October 2011 (week 43) on the website of the Norwegian Institute of Public Health. At that time the use of *Mycoplasma* antibiotics in some counties had already exceeded by three times the use in non-epidemic periods.

Data for three counties from the two regional microbiological laboratories shows that the increase in the number of positive samples from the laboratories for the 6–12 years age group coincided in time with the increase in prescription data (Fig. 4). There is no exact limit for when an epidemic is established, but if one assumes that a steep increase in the number of positive samples would create an alert, the counties in the southeast (Vestfold and Telemark) reached an epidemic level in weeks 40 and 41, respectively, and the county in mid-Norway (Sør-Trøndelag) in week 41. By using the same approach for antibiotic prescribing (steep increase compared to non-epidemic seasons) the level for alert would have been in weeks 40, 41 and 42 for the counties Vestfold, Telemark and Sør-Trøndelag, respectively.

## DISCUSSION

A large increase in number of persons being prescribed erythromycin, clarithromycin and doxycycline was observed in the winter season of 2011–2012, while the use of other antibiotics was quite stable [8]. The increase was associated with an extensive *M. pneumoniae* epidemic identified through the Norwegian voluntary laboratory-based reporting system [8]. *M. pneumoniae* outbreaks are a major cause of increased use of macrolides and tetracyclines, which adds to the risk of development of antimicrobial resistance. In Norway, macrolides and tetracyclines are regarded as broad-spectrum antibiotics compared to penicillins, which are the recommended empirical choice for respiratory tract infections [4]. To limit unnecessary prescribing of macrolides and tetracyclines there is a need to develop strategies to advise prescribers on appropriate antimicrobial practice during and after epidemics.

The voluntary laboratory-based surveillance system is best suited to identify the onset of an epidemic but will not give the full epidemic picture, and it is not designed to follow the epidemic from start to finish. When an epidemic is identified and established, both physicians and laboratories will be reluctant to continue seeking the aetiology to conserve resources. Moreover, the accuracy of the national data is hampered by the fact that the different laboratories are not unanimous in their use of diagnostic test tools and they apply different procedures to handle samples. Some laboratories apply diagnostic panels of multiple respiratory pathogens, while other laboratories test for the requested microbe only (e.g. *M. pneumoniae*). Because of these dissimilarities, general geographical comparisons concerning the magnitude of the epidemic cannot be performed by only using laboratory data.

There is no exact limit defining when an epidemic is established. However, when the proportion of positive samples increases from the background level, the microbial laboratory will send out a signal. The lag time for reporting will depend on the magnitude of positive samples received and whether the epidemic is known, e.g. in neighbouring areas. Utilizing signals from a drug register may add to and strengthen the reports from the laboratory system. We have shown that increased antibiotic prescribing coincides in time with the increase in positive samples. The benefit of using a nationwide drug database is that the whole population is covered and that the epidemic can be mapped throughout the country. Drug use data are reported through the pharmacy data system and transferred to the national database at the beginning of each month. Changes and fluctuations in use will therefore become visible from 15 to 45 days after the drug is being prescribed, depending on whether the prescription was purchased from the pharmacy at the beginning or at the end of the month. Although there is a lag time, the information will allow the public health authorities an opportunity to inform prescribers when the epidemic is fading. A limitation of the drug database is that diagnoses are not included; therefore, an increase in antibiotic use is not unambiguous, but it is a signal that something is occurring.

A common problem in primary care is the lack of verified microbiological diagnoses at the start of therapy. Knowledge of an ongoing epidemic may change physicians' prescribing behaviour. Appropriate feedback from authorities can potentially secure rapid reversion to normal prescribing behaviour once the epidemic is over. The surveillance of antibiotic use makes it possible to detect inappropriate patterns of use and, as such, it is an important part of antibiotic stewardship programmes [5, 6, 15–17]. Our results show that routine data from drug registers can be used to follow the progress of an established epidemic. This way of using drug statistics have seldom been studied and are – to our knowledge – not yet applied in clinical practice. Over-the-counter (OTC) medication sales have been used to study outbreaks of influenza seasons and anthrax [18–20], but OTC sales have been shown to be difficult to use as an indicator because of low specificity of data and problems in data collection. Medication sales data have been studied in France to identify outbreaks of gastroenteritis [21] and the reporting of the 2011 *M. pneumoniae* epidemic in Sweden and Norway was supported by general drug use data [8, 22, 23]. We could not find any studies using information from full-coverage drug databases for syndrome surveillance. By the use of information from the drug databases the health authorities can promote appropriate prescribing of antibiotics connected to outbreaks. This could be done by notifying prescribers, for example the information that an epidemic has faded out in an area may act as guidance for rational antibiotic prescribing, and lead to less prescribing of broad-spectrum antibiotics.

Children aged 6–12 years were chosen as an indicator group for the identification of *M. pneumoniae* epidemics. This age group normally has little antibiotic use and, an increase in use is more easily detectable compared to a high-use population, where the fluctuations will be smaller. Thus, despite the fact that children are more prone to have an asymptomatic infection [3] this age group is probably an optimal indicator group for *M. pneumoniae*.

Because of a generally low occurrence of antibiotic resistance in Norway penicillins are the recommended drugs of choice for bacterial respiratory infections and phenoxymethylpenicillin is most frequently used. Macrolides are used to treat infections caused by *M. pneumoniae, Chlamydophila pneumoniae* and *B. pertussis* but are also recommended in the case of penicillin allergy [12]. A sudden shortage of erythromycin was announced to Norwegian physicians through a ‘dear doctor’ letter from the Norweg-ian Medicines Agency on 3 January 2012 [24]. This probably caused the observed delayed waves of doxycycline and clarithromycin. Tetracyclines are contraindicated to children aged <12 years, and therefore their use was expected to be small or non-existent. However, drug data were collected for the three defined *Mycoplasma* antibiotics for all ages and, interestingly, we also found increased use of tetracyclines in children during the epidemic period, more so for the upper ages of the 6–12 years age group (data not shown).

A limitation of this study is the lack of denominator data for the microbiological samples. We were therefore not able to determine the positivity rate of infection of microbiological samples. The epidemic peak was during the winter months when other respiratory infections are common; however, all other antibiotics followed a normal seasonal pattern, leaving the epidemic to be the most obvious cause. Therapy traditions differ in counties, but the differences are stable, hence for the purpose of syndromic surveillance, we should use correction factors for what is ‘current therapy’ in the actual area. If, however, other infections are epidemic in the same period, e.g. *B. pertussis*, we will not be able to distinguish the two epidemics by using only the NorPD for surveillance. In our study, there was an increased number of positive samples for *B. pertussis* in 2011 [13]. However, increased *B. pertussis* activity is also reported in years without *M. pneumoniae* epidemics (e.g. 2002 and 2009) and without much increase in broad-spectrum antibiotics (data not shown). The increased number of positive samples of *B. pertussis* reported during *M. pneumoniae* epidemics could be explained by the standard procedures at some laboratories, i.e. sending the sample through a standard test panel, thereby identifying more *B. pertussis* cases. Both the NorPD and the voluntary laboratory system captured the epidemic. Data from the laboratories are not equipped to say enough about the extent of the epidemic, and drug use data cannot identify the cause. Microbiological laboratories can provide information about when the epidemic begins and the types of microbes. However, at the national level the current laboratory-based reporting in Norway is limited by a time lag. The NorPD adds value to surveillance of epidemics by systematic and timely collection of data at the national level, and may be useful for monitoring the further development of the epidemic until it levels out and finally ends. Data from multiple sources can be used to give a more complete description of the epidemic, to increase knowledge and to improve quality of surveillance. By establishing a systematic interaction and analysis of data between the two monitoring systems there is the potential for improved infection control and enhanced prudent antibiotic prescribing in Norway.
